# High Levels of Zinc Affect Nitrogen and Phosphorus Transformation in Rice Rhizosphere Soil by Modifying Microbial Communities

**DOI:** 10.3390/plants11172271

**Published:** 2022-08-31

**Authors:** Haihan Lv, Chenchen Ji, Jingli Ding, Lu Yu, Hongmei Cai

**Affiliations:** 1 Microelement Research Center, Huazhong Agricultural University, Wuhan 430070, China; 2 College of Resources and Environment, Huazhong Agricultural University, Wuhan 430070, China

**Keywords:** excessive Zn, N and P transformation, enzyme activity, microbial community, rice rhizosphere soil

## Abstract

Due to global industrialization in recent decades, large areas have been threatened by heavy metal contamination. Research about the impact of excessive Zn on N and P transformation in farmland has received little attention, and its mechanism is still not completely known. In this study, we planted rice in soils with toxic levels of Zn, and analyzed the plant growth and nutrient uptake, the N and P transformation, enzyme activities and microbial communities in rhizosphere soil to reveal the underlying mechanism. Results showed high levels of Zn severely repressed the plant growth and uptake of N and P, but improved the N availability and promoted the conversion of organic P into inorganic forms in rice rhizosphere soil. Moreover, high levels of Zn significantly elevated the activities of hydrolases including urease, protease, acid phosphatase, sucrase and cellulose, and dehydrogenase, as well as the abundances of *Flavisolibacter*, *Sphingomonas*, *Gemmatirosa*, and *subgroup_6*, which contributed to the mineralization of organic matter in soil. Additionally, toxic level of Zn repressed the nitrifying process by decreasing the abundance of nitrosifying bacteria *Ellin6067* and promoted denitrification by increasing the abundance of *Noviherbaspirillum*, which resulted in decreased NO_3_^−^ concentration in rice rhizosphere soil under VHZn condition.

## 1. Introduction

Due to the global industrialization in recent decades, large areas have been threatened by various pollutants. Particularly, the contamination of heavy metals in agricultural soil was much more pronounced near mining and smelting sites [1,2]. Zinc (Zn) is one of the most abundant trace elements on earth, and Zn concentration in the uplayer soil is increasing faster in recent years [1,3]. Zn content in soil depends on both natural conditions and anthropogenic activities. The natural conditions affecting soil Zn content mainly include soil formation processes, humus content, the capacity of soil sorption and oxidation-reduction, as well as the type of grown plants [4,5]. While the anthropogenic activities enhancing soil Zn content mainly include mining, smelting, electroplating, sewage sludge, petroleum refineries, manure and mineral fertilizers, as well as herbicide, insecticide, and plant growth stimulating chemicals used in agricultural activities [1,6,7,8]. Because Zn is a highly mobile metal element, it is easily absorbed and accumulated by plants. Despite Zn is a necessary element for plant survival and growth, a high level of Zn would induce oxidative damage in cells, decline photosynthesis in leaves, and cause stress symptoms in plants, including stunted seed germination and poor growth, curling and chlorosis young leaves, and ultimately reduced yield production [9].

Excessive Zn in agricultural soil not only threatens the crop growth and yield formation, but also modifies the soil microbiome, which is crucial for soil health and nutrient cycling. Previously, a number of studies have been reported that excessive Zn can disrupt the microbiological balance by reducing microbial biomass, activity, and diversity, as well as affecting the microbial community. For example, the biodiversity of microbial taxon declined by 25% in Zn-contaminated soil with 400 mg/kg Zn when compared to the natural soil with 57 mg/kg Zn [10]. High level of Zn has a strong effect on nitrifying bacteria, symbiotic nitrogen-fixing bacteria, and *Azotobacter* cells [11,12]. Since excessive Zn has direct toxicity to nitrifying bacteria and enzymes involved in nitrification process, the conversion of NH_4_^+^ to NO_3_^−^ can be significantly inhibited by a high level of Zn [12,13]. 

Both nitrogen (N) and phosphorus (P) are crucial for plant survival and growth, as they are the key components of biological molecules, such as proteins, nucleic acids, lipids and chlorophyll [14,15]. More importantly, N and P are also the limiting factors of yield formation for crops in agricultural production [16,17]. In soil, most of the N is present as organic N, which is designated as the SOM (soil organic matter). The SOM is considered as the largest N pool in the soil, and the degradation and mineralization of SOM by microorganisms largely determines the plant growth [18]. N mineralization and nitrification are two crucial N transformation processes and play vital roles in determining soil N availability, and largely driven by multiple bacteria, such as nitrogen-fixing bacteria, nitrifying and denitrifying bacteria, and ammonia oxidizing bacteria [19,20]. In addition to bacteria, fungal abundance is also one of the critical factors driving soil N mineralization and nutrient release, as fungi can secrete extracellular enzymes which contributed to the degradation of recalcitrant organic compounds, such as cellulose and lignin [21]. Similarly, bacteria and fungi play a crucial role in regulating P turnover and P availability in soil, as they can enhance P availability by secreting phytase, phosphatase, protons, organic acids, chelates, and siderophores to mineralize organic P and solubilize inorganic fixed P [22,23]. 

Hence, there might be a strong effect of Zn on N and P availability in soil which is mediated by bacteria and fungi. However, research about the impact of excessive Zn on N and P transformation in farmland has received little attention, and its mechanism is still not completely known. The aim of the present study is to investigate the impact of high levels of Zn on plant growth and nutrient uptake, as well as on the N and P transformation processes in rice rhizosphere soil, and correlate them with the soil enzyme activities, bacteria and fungi communities to reveal the underlying mechanism.

## 2. Results

### 2.1. Plant Growth and Nutrient Uptake of Rice

**Figure 1:**
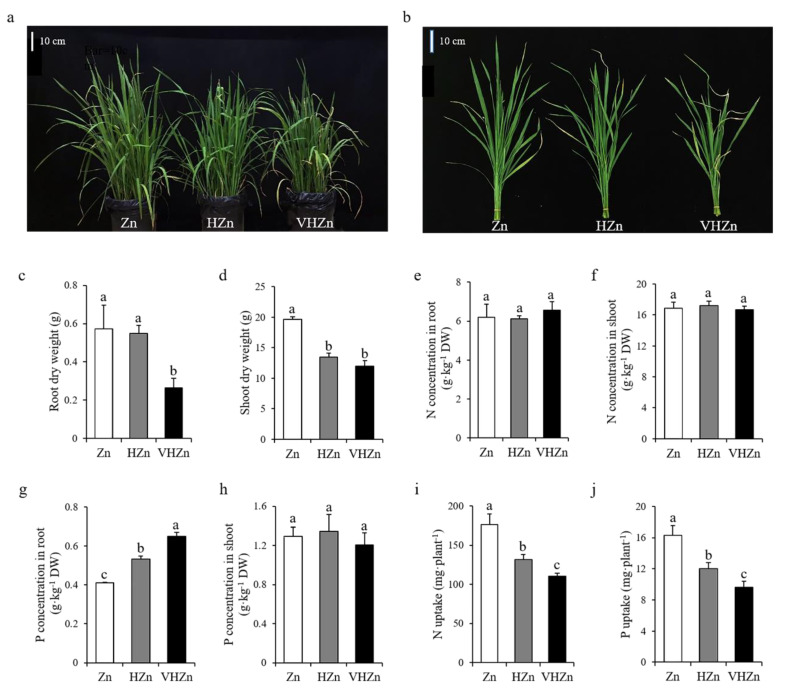
Growth phenotype (**a**,**b**), root and shoot dry weight (**c**,**d**), N concentrations in root and shoot (**e**,**f**), P concentrations in root and shoot (**g**,**h**), N and P uptake (**i**,**j**) of rice plant grown in soil with different levels of Zn (Zn: 10 mg·kg^−1^ soil; HZn: 200 mg·kg^−1^ soil; VHZn: 400 mg·kg^−1^ soil). Data are means ± SD of three biological replicates. Different letters indicate significant difference at *p* < 0.05 by Tukey’s test.

To assess the effect of different levels of Zn on rice growth and nutrient uptake, rice seedlings were grown in soil with the application of 10, 200, and 400 mg·kg^−1^ Zn, and designated as Zn, HZn, and VHZn, respectively. The plant growth was significantly (*p* < 0.05) inhibited by HZn and VHZn treatments compared to Zn treatment, indicating both HZn and VHZn were the toxic levels for rice growth (Figure 1a,b). The plant height, tiller number, leaf SPAD value, root and shoot dry weight all significantly (*p* < 0.05) decreased under HZn and VHZn conditions compared to Zn condition (Figure 1c,d; Supplementary Figure S1). The Zn concentration in both root and shoot, as well as the Zn uptake dramatically increased under HZn and VHZn conditions compared to Zn condition (Supplementary Figure S2). However, no clear changes of N concentrations in root and shoot, or P concentration in shoot were observed, while there was an increase in P concentration in root under HZn and VHZn conditions compared to Zn condition (Figure 1e–h). Different to Zn uptake, the N and P uptake were significantly (*p* < 0.05) declined under HZn and VHZn conditions compared to Zn condition (Figure 1i,j).

### 2.2. Basic Chemical Properties of Rice Rhizosphere Soil

No significant (*p* < 0.05) changes of pH value, organic matter, and available P in rice rhizosphere soil were observed under different Zn conditions (Table 1). The concentrations of available Zn and alkali-hydrolyzed N gradually increased with the increasing levels of Zn treatment, while the available potassium only increased under VHZn condition in the rice rhizosphere soil (Table 1). Compared to Zn condition, the concentration of alkali-hydrolyzed N increased by 32.80% and 68.86% under HZn and VHZn conditions, respectively (Table 1).

### 2.3. Different Forms of N and P in Rice Rhizosphere Soil

**Figure 2:**
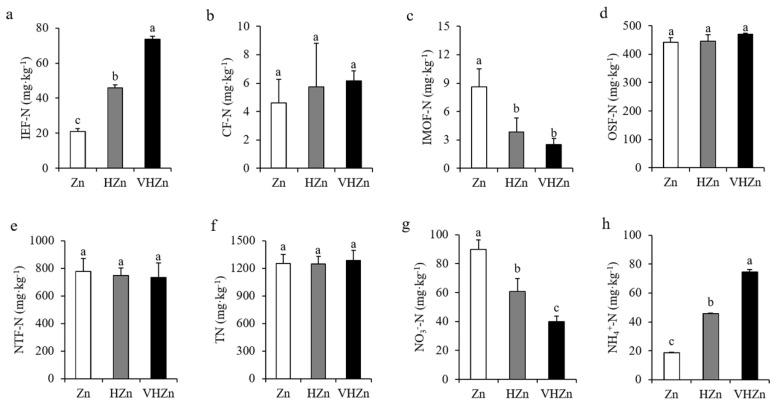
Concentrations of different N forms ((**a**), IEF-N, ion exchanged nitrogen; (**b**), CF-N, carbonate bounded nitrogen; (**c**), IMOF-N, iron-manganese oxide nitrogen; (**d**), OSF-N, organic sulfide bounded nitrogen; (**e**), NTF-N, non-transferable nitrogen; (**f**), T-N, total nitrogen; (**g**), NO_3_^−^, nitrate; (**h**), NH_4_^+^, ammonium) in rice rhizosphere soil with different levels of Zn (Zn: 10 mg·kg^−1^ soil; HZn: 200 mg·kg^−1^ soil; VHZn: 400 mg·kg^−1^ soil). Data are means ± SD of three biological replicates. Different letters indicate significant difference at *p* < 0.05 by Tukey’s test.

To further investigate the effect of different levels of Zn on N and P transformation, we analyzed the different forms of N and P in rice rhizosphere soil. The concentrations of IE-Zn (acid extraction zinc), R-Zn (reducible zinc), and O-Zn (oxidizable zinc) dramatically increased, while the SR-Zn (solid residue Zn) decreased in the rice rhizosphere soil under HZn and VHZn conditions compared to Zn condition (Supplementary Figure S3). Interestingly, high levels of Zn clearly elevated the concentration of IEF-N (ion exchanged nitrogen) and NH_4_^+^-N (ammonium nitrogen), but declined the concentration of IMOF-N (iron-manganese oxide nitrogen) and NO_3_^−^-N (nitrate nitrogen), while did not influence CF-N (carbonate bounded nitrogen), OSF-N (organic sulfide bounded nitrogen), and TN (total nitrogen) in rice rhizosphere soil (Figure 2). The concentration of IEF-N increased 2.18-fold and 3.52-fold, while IMOF-N decreased by 44.87% and 29.49% under HZn and VHZn conditions, respectively, when compared to Zn condition (Figure 2a,c). The concentration of NH_4_^+^-N increased 2.44-fold and 3.97-fold, while NO_3_^−^-N decreased 67.40% and 44.54% under HZn and VHZn conditions, respectively, when compared to Zn condition (Figure 2g,h).

**Figure 3:**
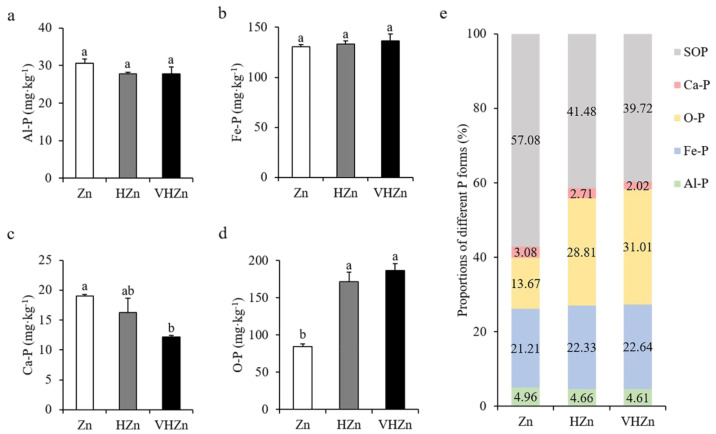
Concentrations (**a**–**d**) and proportions (**e**) of different P forms in rice rhizosphere soil with different levels of Zn (Zn: 10 mg·kg^−1^ soil; HZn: 200 mg·kg^−1^ soil; VHZn: 400 mg·kg^−1^ soil). Al-P, aluminum bound P; Fe-P, iron bound P; Ca-P, calcium bound P; O-P, occluded P; SOP, soil organic P. Data are means ± SD of three biological replicates. Different letters indicate significant difference at *p* < 0.05 by Tukey’s test.

High levels of Zn significantly declined the concentration of Ca-P (calcium bound P), but elevated the concentration of O-P (occluded P), while did not affect Al-P ((aluminum bound P) and Fe-P (iron bound P) in rice rhizosphere soil (Figure 3a–d). The concentrations of Ca-P decreased 85.46% and 64.12%, while O-P increased 2.04-fold and 2.22-fold under HZn and VHZn conditions, respectively, when compared to Zn condition (Figure 3c,d). Then, we calculated the proportions of each form of P including soil organic P (SOP) in rice rhizosphere soil. Results clearly showed the proportion of soil organic P decreased 15.60% and 17.36% under HZn and VHZn conditions, respectively, when compared to Zn condition (Figure 3e), which suggested high levels of Zn could facilitate the conversion of organic P to inorganic P in rice rhizosphere soil.

### 2.4. Enzyme Activities in Rice Rhizosphere Soil

**Figure 4:**
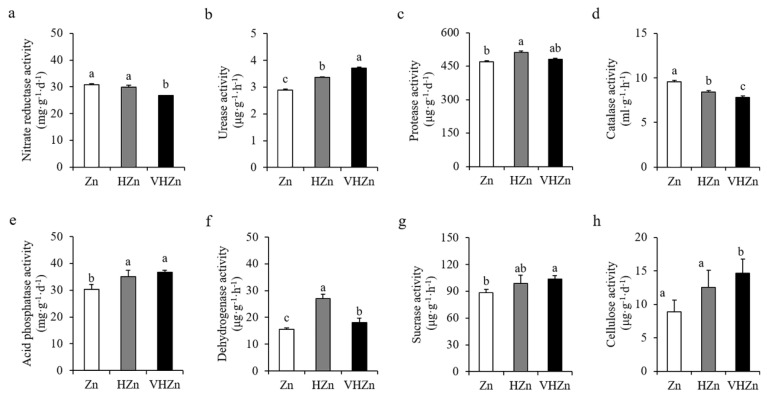
Activities of key enzymes in rice rhizosphere soil with different levels of Zn (Zn: 10 mg·kg^−1^ soil; HZn: 200 mg·kg^−1^ soil; VHZn: 400 mg·kg^−1^ soil). (**a**), Nitrate reductase activity; (**b**), Urease activity; (**c**), Protease activity; (**d**), Catalase activity; (**e**), Acid phosphatase activity; (**f**), Dehydrogenase activity; (**g**), Sucrase activity; (**h**), Cellulose activity. Data are means ± SD of three biological replicates. Different letters indicate significant difference at *p* < 0.05 by Tukey’s test.

Soil enzymes are the key indicators for nutrient cycling in soil. Therefore, we determined the activities of various enzymes in the rice rhizosphere soil with different levels of Zn. Results showed high levels of Zn significantly (*p* < 0.05) increased the activities of urease, protease, acid phosphatase, dehydrogenase, sucrase, and cellulose, while decreased the activities of nitrate reductase and catalase in rice rhizosphere soil (Figure 4). The activity of urease increased 16.26% and 28.37%, protease increased 9.03% and 2.51%, acid phosphatase increased 15.61% and 20.88%, dehydrogenase increased 73.48% and 15.63%, sucrase increased 11.38% and 17.20%, and cellulose increased 41.28% and 65.13%, while the activity of nitrate reductase decreased 96.98% and 86.70%, and catalase decreased 88.22% and 81.94% under HZn and VHZn conditions, respectively, when compared to Zn condition (Figure 4).

### 2.5. Bacterial Communities in Rice Rhizosphere Soil

16S rRNA sequencing was used to reveal the effect of different levels of Zn on the alpha diversity of bacterial communities in the rice rhizosphere soil (Supplementary Figure S4). Both Simpson and Pielou_e indices showed that high levels of Zn significantly (*p* < 0.05) increased the bacterial diversity in rice rhizosphere soil (Supplementary Figures S4 and S5a). However, no significant (*p* < 0.05) differences were observed in other indices, including Chao 1, Faith_pd, Goods_coverage, Shannon, and Observed_species, under different Zn conditions (Supplementary Figure S4). 1511, 1776, and 1480 unique OTUs were found in the rice rhizosphere soil under Zn, HZn, and VHZn conditions, respectively (Supplementary Figure S5b). The PCoA (principal coordinate analysis) analysis showed that bacterial communities from different Zn treatments were clearly separated into distinct clusters along the X-axis (Supplementary Figure S5c). 

**Figure 5:**
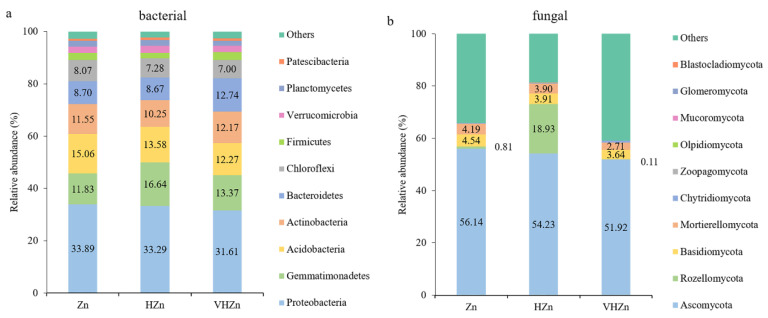
Relative abundances of the top classified phyla of bacterial (**a**) and fungal (**b**) communities in rice rhizosphere soil with different levels of Zn (Zn: 10 mg·kg^−1^ soil; HZn: 200 mg·kg^−1^ soil; VHZn: 400 mg·kg^−1^ soil).

The most abundant bacterial phyla were selected to monitor changes in the bacterial community among different Zn treatments. *Proteobacteria*, followed by *Gemmatimonadetes*, *Acidobacteria*, *Actinobacteria*, *Bacteriodetes*, and *Chloroflexi* were the six most abundant phyla across all treatments among the classified bacteria phyla, accounting for ~80% of all bacteria sequences (Figure 5a). However, the abundance of bacteria phyla did not change much among different Zn treatments, except the abundance of *Gemmatimonadetes* increased 4.81% under HZn condition, when compared to Zn condition (Figure 5a). 

**Figure 6:**
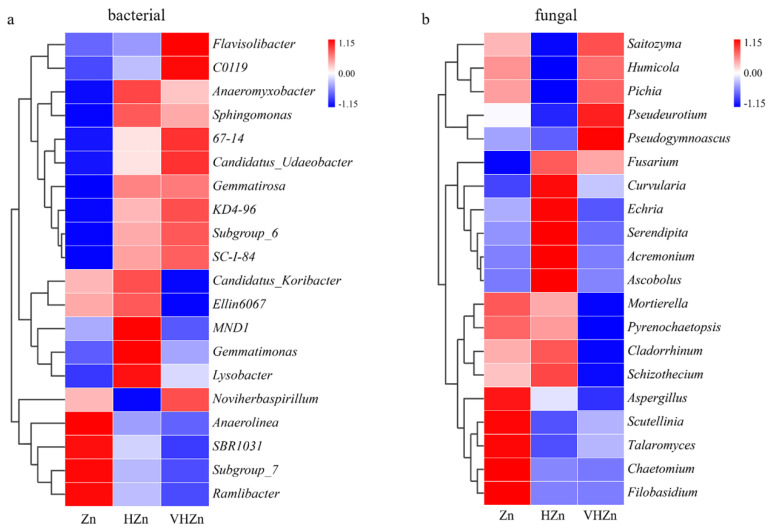
Hierarchical clustering heat maps of the bacterial (**a**) and fungal (**b**) communities in top 20 genera in rice rhizosphere soil with different levels of Zn (Zn: 10 mg·kg^−1^ soil; HZn: 200 mg·kg^−1^ soil; VHZn: 400 mg·kg^−1^ soil).

A heatmap generated from the top 20 classified bacteria genera displayed diverse changes among different Zn treatments. The abundances of *Flavisolibacter*, *C0119*, *Anaeromyxobacter*, *Sphingomonas*, *67-14*, *Candidatus_Udaeobacter*, *Gemmatirosa*, *KD4-96*, *Subgroup_6*, and *SC-1-84* increased, while the abundances of *Anaerolinea*, *SBR1031*, *Subgroup_7*, and *Ramlibacter* decreased under both HZn and VHZn conditions, when compared to Zn condition (Figure 6a). The abundances of *Candidatus_Koribacter*, *Ellin6067*, *MND1*, *Gemmatimonas*, and *Lysobacter* increased under HZn condition, while decreased under VHZn condition, when compared to Zn condition (Figure 6a). Only the abundance of *Noviherbaspirillum* decreased under HZn condition, while increased under VHZn condition, when compared to Zn condition (Figure 6a). 

### 2.6. Fungal Communities in Rice Rhizosphere Soil

ITS sequencing was used to reveal the effect of different levels of Zn on the alpha diversity of fungal communities in the rice rhizosphere soil. No significant (*p* < 0.05) differences were observed in Chao 1, Observed_species, Shannon, Simpson, or Pielou_e indices under different Zn conditions, while only the Goods_coverage index significantly (*p* < 0.05) increased under HZn condition compared to Zn condition. 657, 678, and 725 unique OTUs were found in the rice rhizosphere soil with Zn, HZn, and VHZn treatments, respectively. The PCoA analysis showed that fungal communities from different Zn treatments were distinct along both *X*-axis and *Y*-axis.

The most abundant fungal phyla were selected to monitor changes in the fungal community among different Zn treatments. *Ascomycota*, *Rozellomycota*, *Basidiomycota*, and *Mortierellomycota* were the four most abundant phyla across all treatments among the classified fungal phyla (Figure 5b). The abundance of *Rozellomycota* dramatically increased by 18.12% under HZn condition, while only decreased by 0.70% under VHZn condition, when compared to the Zn condition (Figure 5b). However, the abundances of *Ascomycota*, *Basidiomycota*, and *Mortierellomycota* all slightly decreased with the increasing level of Zn treatment (Figure 5b). 

A heatmap generated from the top 20 classified fungal genera displayed diverse changes among different Zn treatments. The abundances of *Fusarium* and *Curvularia* increased, while the abundances of *Mortierella*, *Pyrenochaetopsis*, *Aspergillus*, *Scutellinia*, *Talaromyces*, *Chaetomium*, *Filobasidium* decreased under both HZn and VHZn conditions, when compared to Zn condition (Figure 6b). The abundances of *Echria*, *Cladorrhinum*, and *Schizothecium* increased under HZn condition, while decreased under VHZn condition, when compared to Zn condition (Figure 6b). The abundances of *Saitozyma*, *Humicola*, *Pichia*, *Pseudeurotium*, and *Pseudogymnoascus* decreased under HZn condition, while increased under VHZn condition, when compared to Zn condition (Figure 6b). The abundances of *Serendipita*, *Acremonium,* and *Ascobolus* only increased under HZn condition when compared to Zn condition (Figure 6b). 

### 2.7. Correlations between Microbial Communities and Soil Characteristics 

**Figure 7:**
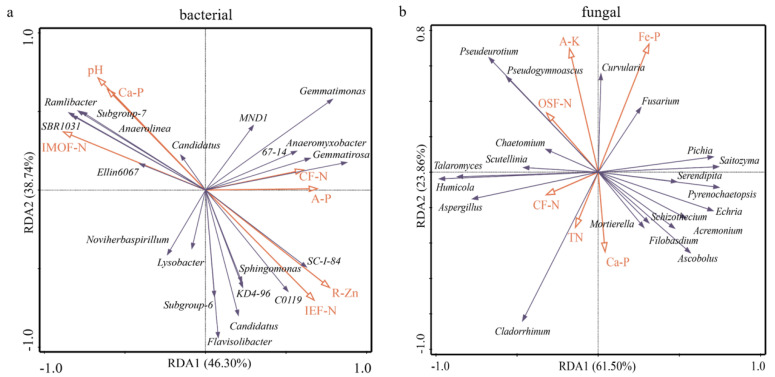
Redundancy analysis (RDA) of bacterial (**a**) and fungal (**b**) communities in rice rhizosphere soil. RDA correlation plot showed variance in genera of bacterial and fungal explained by soil chemical factors. EF-N, ion exchanged nitrogen; CF-N, carbonate bounded nitrogen; IMOF-N, iron-manganese oxide nitrogen; OSF-N, organic sulfide bounded nitrogen; T-N, total nitrogen; A-P, available P; Fe-P, iron bound P; Ca-P, calcium bound P; A-K, available potassium; R-Zn, solid residue zinc.

An RDA (redundancy analysis) analysis was used to explain the possible correlation between soil characteristics and microbial communities in rice rhizosphere soil (Figure 7). Results showed the bacterial community was mainly correlated with pH, IMOF-N, CF-N, IEF-N, Ca-P, A-P (available P), and R-Zn, while the fungal community was more correlated with OSF-N, CF-N, TN, Ca-P, Fe-P, and A-K (available potassium) (Figure 7). For example, the bacterial genera *SBR 1031*, *Ramlibacter*, *Subgroup_7*, and *Anaerolinea* were positively affected by pH, IMOF-N, and Ca-P, but negatively affected by IEF-N and R-Zn, the *Gemmatimonas*, *Anaeromyxobacter*, and *Gemmatirosa* were positively affected by CF-N and A-P, while the *SC-1-84*, *C0119*, *Sphingomonas*, *KD4-96*, and *Candidatus* were positively affected by IEF-N and R-Zn, but negatively affected by pH, IMOF-N, and Ca-P (Figure 7a). The A-K and OSF-N positively affected fungal genera *Pseudeurotium* and *Pseudogymnoascus*, Fe-P positively affected *Curvularia* and *Fusarium*, while CF-N, TN, and Ca-P positively affected *Cladorrhinum* (Figure 7b).

**Figure 8:**
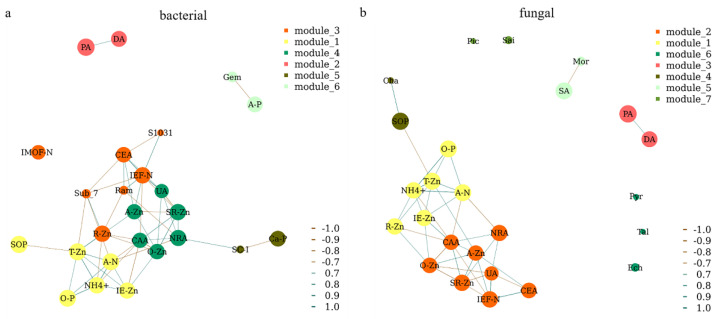
Network analysis of the bacterial (**a**) and fungal (**b**) genera, enzyme activities, and different forms of Zn, N, and P in rice rhizosphere soil. A correlation coefficient threshold of Spearman’s *r_s_* ≥ 0.9 with *p* value ≤ 0.05 was used. A-N, alkali-hydrolyzed nitrogen; A-P, available phosphorus; A-Zn, available zinc; IE-Zn, acid extraction zinc; R-Zn, reducible zinc; O-Zn, oxidizable zinc; SR-Zn, solid residue zinc; T-Zn, total zinc; IMOF-N, iron-manganese oxide nitrogen; IEF-N, ion exchanged nitrogen; NH_4_^+^, ammonium; O-P, occluded phosphorus; Ca-P, calcium bound phosphorus; SOP, soil organic phosphorus; PA, protease activity; UA, urease activity; DA, dehydrogenase activity; SA, surase activity; CAA, catalase activity; NRA, nitrate reductase activity; CEA, cellulose activity; Gem, *Gemmatimonas*; Sub_7, Subgroup_7; Ram, *Ramlibacter*; SC-I, SC-I-84; S1031, SBR1031; Mor, *Mortierella*; Ech, *Echria*; Sai, *Saitozyma*; Cha, *Chaetomium*; Pyr, *Pyrenochaetopsis*; Ser, *Serendipita*.

A network analysis was further performed to show the close relationships between microbial genera, enzyme activities, and different forms of N, P, and Zn in rice rhizosphere soil (Figure 8). A strong relationship was observed between the soil enzyme activities (UA, NRA, and CAA) and different forms of Zn (A-Zn, O-Zn, and SR-Zn). Different forms of Zn (T-Zn, R-Zn, and IE-Zn) also had strong effects on both N (A-N and NH_4_^+^) and P (O-P and SOP). The bacterial genera *Ram* and *S1031* were positively correlated with IEF-N, while the *Sub_7* was negatively correlated with IEF-N (Figure 8a). The bacterial genera *Gem* and *SC-I* were negatively correlated with A-P and Ca-P, respectively (Figure 8a). The fungal genera *Cha* and *Mor* were positively correlated with SOP and SA, respectively (Figure 8b). There was a positive relationship between PA and DA (Figure 8). However, IMOF, *Pic*, *Sai*, *Pyr*, *Tal*, and *Ech* were individually existed in the network, which means there is no correlations among themselves or other soil characteristics (Figure 8).

## 3. Discussion

### 3.1. High Levels of Zn Improved N Availability in Rice Rhizosphere Soil

N is a major essential nutrient for plant growth. The N availability in soil is a limiting factor for plant N uptake, and it determines the growth and yield production of crops in agricultural system [17,24]. Although it has been reported there is a synergistic effect of Zn on the N turnover in soils, as it serves as a cofactor for many enzymes involved in N metabolism [25], whether the toxic level of Zn still has positive effect on N transformation in soil receives little attention. In the present study, we found high levels of Zn clearly elevated the concentration of IEF-N, alkali-hydrolyzed N, and NH_4_^+^-N, but declined the concentration of IMOF-N and NO_3_^−^-N in rice rhizosphere soil (Table 1, Figure 2). These results indicated even the toxic level of Zn could facilitate the N turnover and improve the N availability in soil. Among the various forms of N, NO_3_^−^ is the main source in the majority of agricultural soils, and is the ideal N form taken up by most crops, while rice is more likely to uptake NH_4_^+^ from the paddy soil [26,27]. Here, we found high levels of Zn significantly increased the concentration of NH_4_^+^, but decreased the concentration of NO_3_^−^ in rice rhizosphere soil (Figure 2g,h), which seems to be an advantage for rice growth and N uptake. However, no clear changes in N concentration of root and shoot were observed, and the N uptake of rice plant significantly decreased due to the declined plant biomass under high levels of Zn (Figure 1a–f,j). Therefore, the inhibition of plant growth under high levels of Zn may be due to the biochemical toxicity of Zn in plant cells, such as inducing a large amount of reactive oxygen species and causing oxidative stress, but not because of the N availability in soil or the N uptake ability of plant.

### 3.2. High Levels of Zn Promoted the Conversion of Organic P into Inorganic P

In soil, various forms of P exist with different availabilities, but only a very small fraction of total P, about 0.1%, is available to plants [28,29]. Therefore, P availability in soil is also one of the critical limiting factors for crop growth and grain production in agriculture. Here, we did not find any change in the concentration of available P in rice rhizosphere soil under different levels of Zn (Table 1). Moreover, high levels of Zn significantly declined the concentration of Ca-P, but elevated the concentration of O-P (Figure 3c,d), which indicated high levels of Zn did not have any positive effect on P availability in rice rhizosphere soil. Although inorganic P is considered as a major source of available P to plants, organic P is a large P pool and plays an important role in P cycling, as it comprises 30–65% of total P in soil [30,31]. After we calculated the proportions of each form of P including organic P in rice rhizosphere soil, we found high levels of Zn significantly reduced the proportion of soil organic P by 15.60–17.36% (Figure 3e), which suggested high levels of Zn could facilitate the conversion of organic P to inorganic P in rice rhizosphere soil. Unexpectedly, the P concentration clearly increased in root, while did not change in shoot under high levels of Zn (Figure 1g,h). Similar to N, the P uptake of rice plant significantly decreased under high levels of Zn (Figure 1j). The detailed mechanism in rice plant needs to be further investigated.

### 3.3. High Levels of Zn Altered Enzyme Activities in Rice Rhizosphere Soil

As we know, soil enzyme is a sensitive indicator for nutrient cycling in soil [32,33]. Heavy metals have direct or indirect effect on soil enzyme activities, as they would directly change the activity of free and extracellular enzymes or indirectly affect the intracellular enzyme biosynthesis by influencing both microorganisms in soil and root exudation from plant [34]. Previous reports showed excessive amount of Zn could disrupt the soil enzyme activities involved in C, N, P, and S cycling [35,36]. Here, our results showed high levels of Zn significantly increased the activities of urease, protease, acid phosphatase, hydrogenase, sucrase, and cellulose, while decreased the activities of nitrate reductase and catalase in rice rhizosphere soil (Figure 4). These results are quite similar to the studies performed by Yang et al., which revealed a positive relationship between Zn and urease, acid phosphatase, and catalase [35]. The higher activities of hydrolases in our study including urease, protease, acid phosphatase, sucrase and cellulose, and dehydrogenase mainly contribute to the transformation of N and P from organic matter into inorganic forms, which finally improves the N availability in rice rhizosphere soil under high levels of Zn. The decreased activity of nitrate reductase under VH Zn condition may be due to the higher NH_4_^+^ concentration in rice rhizosphere soil for the feedback regulation. Furthermore, the decreased activity of catalase under high levels of Zn may increase the risk from reactive oxygen species in rice rhizosphere soil, which would be harmful for both microorganisms and plant growth.

### 3.4. High Levels of Zn Modified the Bacterial and Fungi Communities

The communities and abundances of microorganisms are the crucial factors driving N and P cycling in soil, as they are effective at releasing N and P from soil and increasing their availability to plants [19,20,37]. Previous studies showed high level of Zn has a strong effect on nitrifying bacteria, symbiotic nitrogen-fixing bacteria, and *Azotobacter* cells [11,12]. In this study, we found high levels of Zn elevated the abundances of *Flavisolibacter*, *Sphingomonas*, *Gemmatirosa, and subgroup_6*, which are involved in the degradation of biomacromolecules and biopolymers (Figure 6a). It is well-known that the genus *Sphingomonas* has the ability to degrade aromatic and xenobiotic compounds [38]. Several species of *Sphingomonas* have been identified as N-fixing bacteria, such as *Sphingomonas azotifigens* and *Sphingomonas paucimobilis* [39,40]. Moreover, Videira et al. have isolated 22 *Sphingomonas*-like species from two rice varieties grown in soil and identified them as N-fixing bacteria [41]. Therefore, our results suggested high levels of Zn facilitated the mineralization process of N and P, promoting the nutrient turnover to inorganic forms from organic matters in rice rhizosphere soil. Interestingly, the abundance of *Ellin6067* which is designated as the nitrosifying bacteria decreased, and the abundance of *Noviherbaspirillum* which is designated as the denitrifying bacteria [42] increased under VH Zn condition (Figure 6a), indicating toxic level of Zn repressed the nitrifying process preventing NH_4_^+^ losing and promoted the denitrification, which resulted in the decreasing of NO_3_^−^ but increasing of NH_4_^+^ in the rice rhizosphere soil (Figure 2g,h). Similar results have been reported by previous studies that excessive Zn significantly inhibited the nitrification process and the conversion of NH_4_^+^ to NO_3_^−^ [12,13]. Members of the genus *Mortierella* are filamentous, lignocellulose decomposing fungi, which can be found on almost any substrate and are often encountered in soils as saprophytes [43]. *Mortierella* species can produce polyunsaturated fatty acids, as well as arachidonic, γ-linolenic, eicosapentaenoic, and docosahexaenoic acids in the mycelium by converting excess of carbon sources into lipids under different fermentation conditions [44,45,46]. These compounds produced by *Mortierella* are involved in the induction of resistance to phytopathogens in plants [47]. Here, we found that high levels of Zn significantly decreased the relative abundance of *Mortierella* (Figure 6b). Moreover, we found high levels of Zn also elevated the abundances of pathogenic fungi *Fusarium*, *Curvularia*, and *Ascobolus* in rice rhizosphere soil (Figure 6b), which maybe one of the reasons for the poor growth of rice under toxic level of Zn. 

## 4. Materials and Methods

### 4.1. Plant Materials and Growth Conditions

Seeds of rice (Guangliangyou 35) were soaked in water for two days and then transferred to a net floating on a 0.5 mM CaCl_2_ solution. After a week, seedlings were planted to pots with 1.0 g N·kg^−1^ soil, 0.15 g P_2_O_5_·kg^−1^ soil, and 0.2 g K_2_O·kg^−1^ soil with different applications of Zn (Zn: 10 mg·kg^−1^ soil; HZn: 200 mg·kg^−1^ soil; VHZn: 400 mg·kg^−1^ soil), and grown at 25 °C to 35 °C under natural light. Three biological replications were conducted. Each replication contains four plants. Eight weeks later, the roots and shoots were harvested for N, P, and Zn determination, and the rhizosphere soils were collected for the analyses of basic chemical properties, different forms of N, P, and Zn, enzyme activities, and microbial communities.

### 4.2. Determination of N, P and Zn in Plant Samples

The root and shoot samples were dried at 65 °C for five days. After recording the dry weight, the samples were ground to a fine powder and subjected to digestion with 5 mL of 98% H_2_SO_4_ and several drops of H_2_O_2_ on a heater at up to 180 °C. The concentrations of total N/P and Zn in the digestion solution were analyzed using a flow injection analyzer (FIAstar 5000, Sweden) and an atomic absorption spectrophotometer (WFX-ID, China), respectively. 

### 4.3. Analyses of Chemical Properties and Enzyme Activities in Soil Samples

The basic chemical properties of rice rhizosphere soil were analyzed according to the methods described by Bao using air-dried soil [48]. The pH value was measured in an extraction of soil: water (1:2.5 *w*/*v*) by a pH meter. The concentration of SOM was measured by the potassium dichromate oxidation method using a 0.136 M K_2_Cr_2_O_7_-H_2_SO_4_ mixture. The concentration of alkaline hydrolysis N in soil was estimated by the alkaline hydrolysis diffusion method using a 1.8 M NaOH solution. The concentration of available P in soil was determined by the molybdenum stibium anti-color method using an extraction of 0.5 M NaHCO_3_. The concentrations of available K and available Zn in soil were estimated by a flame photometer (JK-FP640S, China) using the extraction of 1M NH_4_OAc and 0.005 M DTPA, respectively. The different forms of N, P, and Zn were analyzed as described previously [48,49,50,51]. The enzyme activities in the fresh soil were determined based on the methods described by Guan [52].

### 4.4. DNA Extraction and Microbial Community Analysis of Soil Samples

The microbial DNA was extracted from fresh soil using the OMEGA Soil DNA Kit (Omega Bio-Tek, Norcross, GA, USA). The V4 regions of bacterial 16S rRNA were amplified by forward primer 515F (5′-GTGYCAGCMGCCGCGGTAA-3′) and reverse primer 806R (5′-GGACTACNVGGGTWTCTAAT-3′). The fungal ITS1 regions were amplified by forward primer ITS5F (5′-GGAAGTAAAAGTCGTAACAAGG-3′) and reverse primer ITS1R (5′-GCTGCGTTCTTCATCGATGC-3′). After purifying and normalizing, the PCR products were sequenced. Two paired-end sequencing (2 × 250 bp) was performed on the Illunima Miseq PE250 platform. The sequencing depth is 40,000 for each sample. Microbiome bioinformatics were performed with QIIME2 [53] with slight modification according to the official tutorials (https://docs.qiime2.org/2019.4/tutorials/, accessed on 13 July 2022) [54]. Raw sequence data were demultiplexed using the demux plugin followed by primers cutting with cutadapt plugin. Sequences were then quality filtered, denoised, merged, and chimera removed using the DADA2 plugin. Non-singleton amplicon sequence variants (ASVs) were aligned with mafft and used to construct a phylogeny with fasttree2. Sequences with more than 97% similarity were classified as the same operational taxonomic unit (out). Alpha-diversity metrics including Chao 1, Observed species, Shannon, Simpson, Faith’PD, Pielou’s evenness and Good’s coverage, and beta-diversity metrics including weighted UniFrac, unweight UniFrac, Jaccard distance, and Bray–Curtis dissimilarity were estimated using the diversity plugin. Bacterial and fungal taxonomy were assigned to ASVs using the classify-sklearn naïve Bayes taxonomy classifier in feature-classifier plugin [55] against the SILVA Release 132 and UNITE Release 8.0 Database, respectively [56]. Beta diversity analysis was performed to investigate the structural variation of microbial communities across samples using Bray–Curtis metrics [57] and visualized via principal coordinate analysis (PCoA). Venn diagram was generated to visualize the shared and unique ASVs among samples or groups using R package “VennDiagram”, based on the occurrence of ASVs across samples/groups regardless of their relative abundance [58]. Redundancy analysis (RDA) was performed by Canono 5.02 software to elucidate the relationship between soil physicochemical properties and the abundance of dominant genera of bacteria or fungi. To avoid the overfitting in RDA analysis, the number of selected soil properties are less than the number of total soil samples in this study. To avoid collinearity among variables, we calculated the variance inflation factors (VIFs) in stepwise manner, discarding the soil properties with the highest VIF at each step, until all VIFs were less than 10. The significance test between microbial community compositions and soil properties was evaluated by Envfit test. For the network analysis, the strong and significant correlations (Spearman’s *r_s_* ≥ 0.9 with *p* value ≤ 0.05) among microbial genera, enzyme activities, and different forms of nutrients in rice rhizosphere soil were considered. The network was visualized in Cytoscape 3.5.1 (Shannon, Washington, DC, USA).

### 4.5. Statistical Analysis

SPSS (Statistical Package for the Social Sciences) was used for statistical analysis. Data are presented as means ± SD with three independent biological replicates. Significant differences were determined by Tukey’s test (*p* < 0.05).

## 5. Conclusions

In conclusion, high levels of Zn increased the concentration of alkali-hydrolyzed N, promoted the conversion of IEF-N from IMOF-N, inhibited the transformation of NO_3_^−^ from NH_4_^+^, ultimately improved the N availability in rice rhizosphere soil. However, high levels of Zn only promoted the conversion of P from organic forms into inorganic forms. Since the transformation of O-P from Ca-P was facilitated by high levels of Zn, the concentration of available P in rice rhizosphere soil did not show any changes. Further analysis of enzyme activities and microbial communities well supported these results. Higher activities of hydrolases including urease, protease, acid phosphatase, sucrase and cellulose, and dehydrogenase, as well as increased abundances of *Flavisolibacter*, *Sphingomonas*, *Gemmatirosa, and subgroup_6* were observed under high levels of Zn, which contributed to the transformation of N and P from organic matter into inorganic forms. Additionally, toxic level of Zn repressed the nitrifying process by decreasing the abundance of nitrosifying bacteria *Ellin6067* and promoted denitrification by increasing the abundance of *Noviherbaspirillum*, which resulted in decreased NO_3_^−^ concentration in rice rhizosphere soil under VHZn condition. Despite positive effect of Zn on N and P transformation in rice rhizosphere soil was observed, the N and P uptake was declined in rice plant, because the toxic level of Zn repressed plant growth severely. Therefore, our study analyzed the impact of excessive Zn on N and P transformation in rice rhizosphere soil, and revealed its underlying mechanism mediated by microorganisms.

## Figures and Tables

**Table 1:** Basic chemical properties of rice rhizosphere soil with different levels of Zn supply.

Parameters	Zn	HZn	VHZn
pH value	6.90 ± 0.12 a	6.80±0.09 a	6.70 ± 0.16 a
Organic matter (g·kg^−1^)	11.11 ± 0.17 a	11.22 ± 0.06 a	11.19 ± 0.02 a
Alkali-hydrolyzed nitrogen (mg·kg^−1^)	71.16 ± 4.04 c	94.50 ± 0.54 b	120.16 ± 4.24 a
Available phosphorus (mg·kg^−1^)	15.23 ± 1.37 a	16.97 ± 0.33 a	16.78 ± 1.65 a
Available potassium (mg·kg^−1^)	98.83 ± 5.03 b	101.16 ± 6.80 b	142.83 ± 10.11 a
Available zinc (mg·kg^−1^)	4.71 ± 0.11 c	11.56 ± 0.02 b	15.12 ± 0.03 a

Zn, 10 mg·kg^−1^ soil; HZn, 200 mg·kg^−1^ soil; VHZn, 400 mg·kg^−1^ soil. Data are means ± SD of three biological replicates. Different letters indicate significant difference at *p* < 0.05 by Tukey’s test.

## Data Availability

Not applicable.

## Supplementary Materials

The following supporting information can be downloaded at: https://www.mdpi.com/article/10.3390/plants11172271/s1. Figure S1: Plant height, tiller number, and leaf SPAD value of rice plant grown in soil with different levels of Zn; Figure S2: Zn concentrations in root and shoot, and Zn uptake of rice plant grown in soil with different levels of Zn; Figure S3: Concentrations of different Zn formsin rice rhizosphere soil with different levels of Zn; Figure S4: Alpha diversity indices of bacterial communities in rice rhizosphere soil with different levels of Zn; Figure S5: Simpson index, Venn diagram, and principal coordinate analysis of bacterial communities in rice rhizosphere soil with different levels of Zn.
