# Supplementary material for: High Levels of Zinc Affect Nitrogen and Phosphorus Transformation in Rice Rhizosphere Soil by Modifying Microbial Communities
Source: Plants (Basel). 2022 Aug 31;11(17):2271. doi: 10.3390/plants11172271 (PMC9460630; doi:10.3390/plants11172271)
Supplement: Supplementary file 1 [file plants-11-02271-s001.zip › plants-1841890-supplementary.pdf]

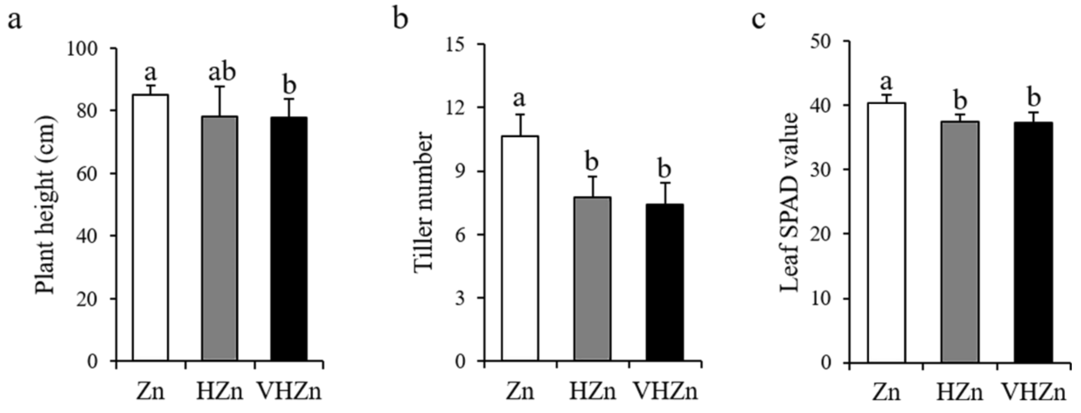

Supplementary Figure S1 Plant height (a), tiller number (b), and leaf SPAD value (c) of rice plant grown in soil with different levels of Zn (Zn: 10 mg/kg soil; HZn: 200 mg/kg soil; VHZn: 400 mg/kg soil). Data are means ±SD of three biological replicates. Different letters indicate significant difference at $P < 0.05$ by Tukey's test.

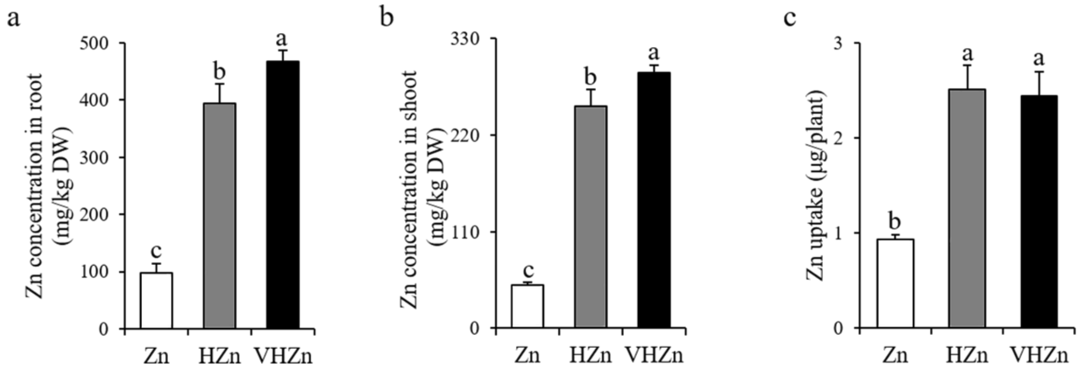

Supplementary Figure S2 Zn concentrations in root and shoot (a, b), and Zn uptake (c) of rice plant grown in soil with different levels of Zn (Zn: 10 mg/kg soil; HZn: 200 mg/kg soil; VHZn: 400 mg/kg soil). Data are means ±SD of three biological replicates. Different letters indicate significant difference at $P < 0.05$ by Tukey's test.

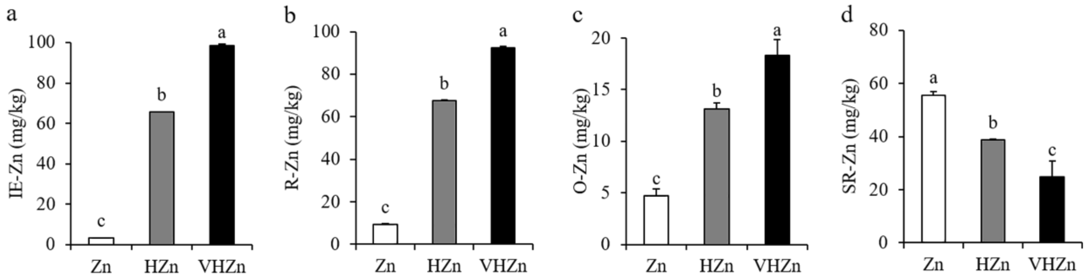

Supplementary Figure S3 Concentrations of different Zn forms (a, IE-Zn, acid extraction zinc; b, R-Zn, reducible zinc; c, O-Zn, oxidizable zinc; d, SR-Zn, solid residue zinc) in rice rhizosphere soil with different levels of Zn (Zn: 10 mg/kg soil; HZn: 200 mg/kg soil; VHZn: 400 mg/kg soil). Data are means ±SD of three biological replicates. Different letters indicate significant difference at $P < 0.05$ by Tukey's test.

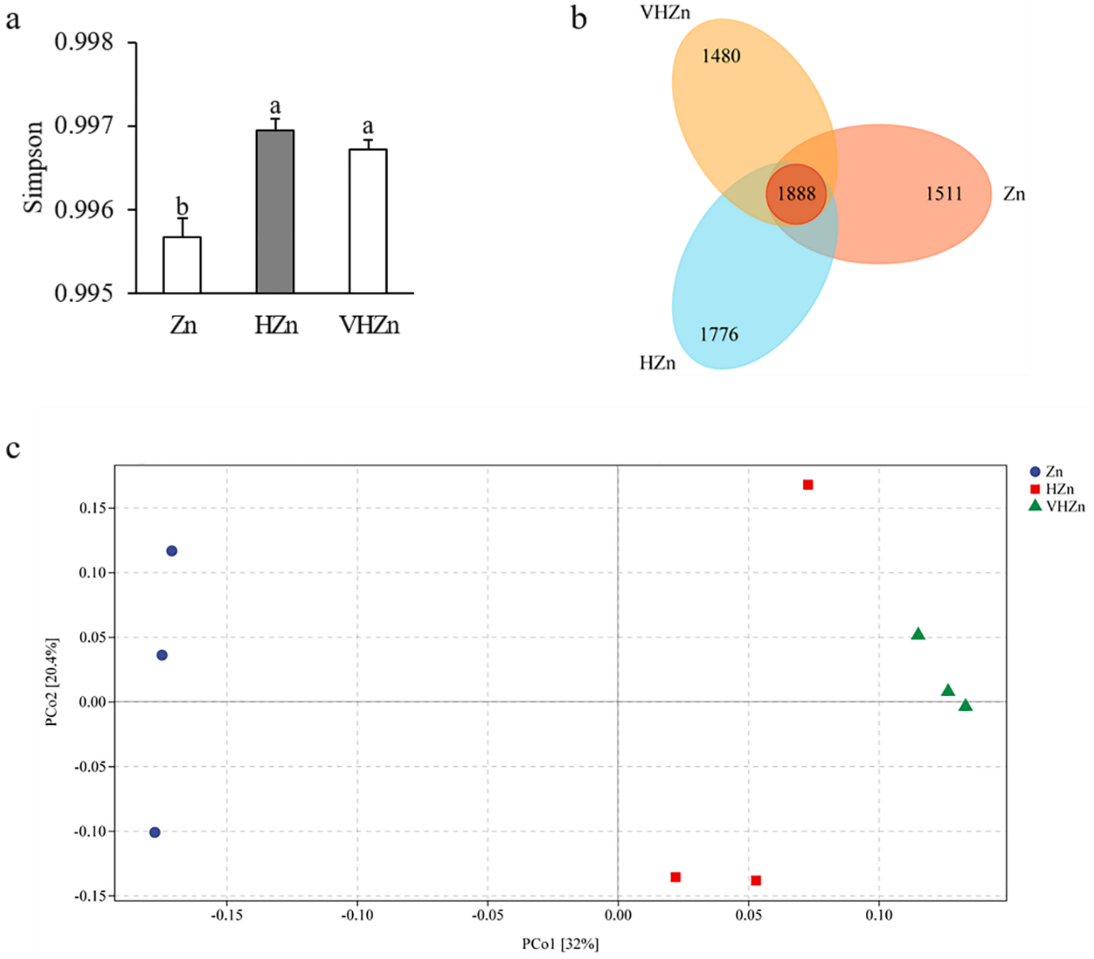

Supplementary Figure S4 Simpson index (a), Venn diagram (b), and principal coordinate analysis (PCoA, c) of bacterial communities in rice rhizosphere soil with different levels of Zn (Zn: 10 mg/kg soil; HZn: 200 mg/kg soil; VHZn: 400 mg/kg soil). Data are means ±SD of three biological replicates. Different letters indicate significant difference at $P < 0.05$ by Tukey's test.

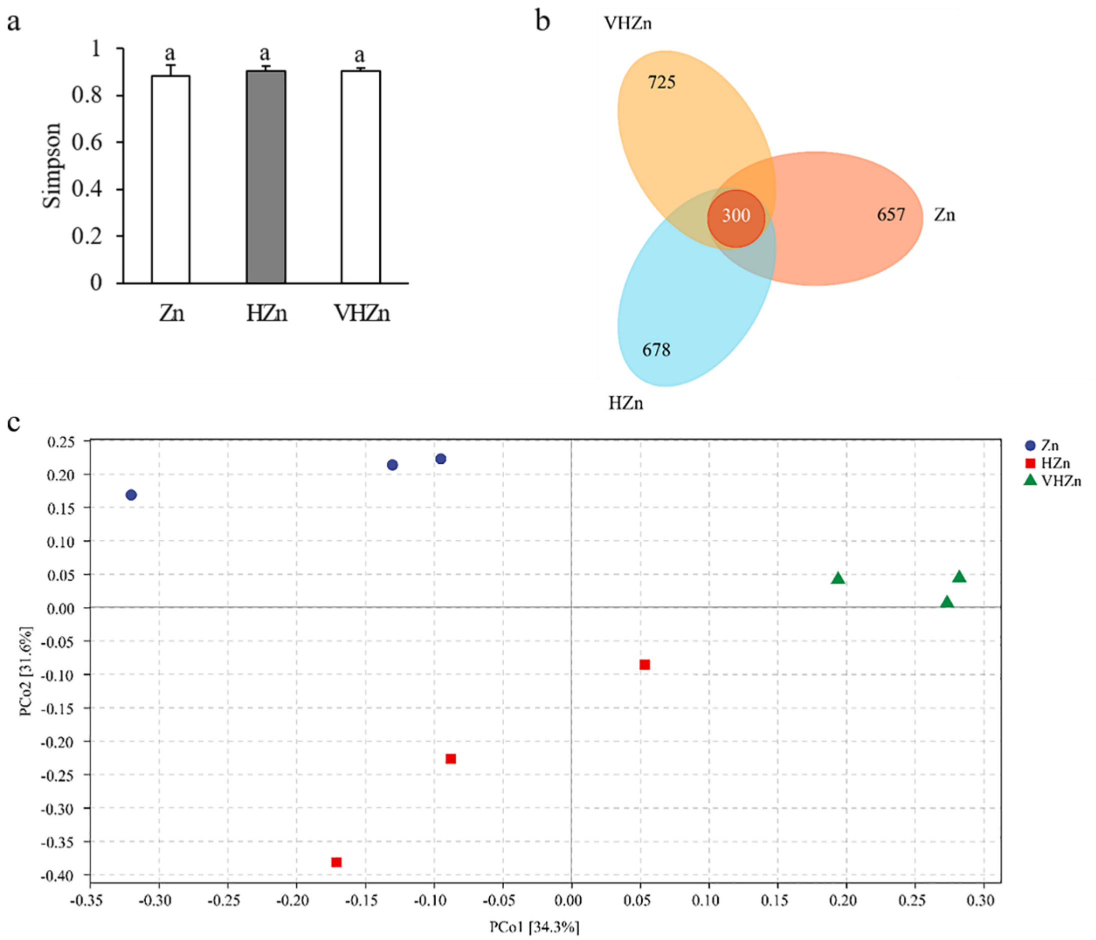

Supplementary Figure S5 Simpson index (a), Venn diagram (b), and PCo analysis (c, principal coordinate analysis) of fungal communities in rice rhizosphere soil with different levels of Zn (Zn: 10 mg/kg soil; HZn: 200 mg/kg soil; VHZn: 400 mg/kg soil). Data are means ±SD of three biological replicates. Different letters indicate significant difference at $P < 0.05$ by Tukey's test.
